# Sulfonated inhibitors of the RNA editing ligases validate the essential role of the MRP1/2 proteins in kinetoplastid RNA editing

**DOI:** 10.1261/rna.075598.120

**Published:** 2020-07

**Authors:** Vaibhav Mehta, Houtan Moshiri, Akshaya Srikanth, Smriti Kala, Julius Lukeš, Reza Salavati

**Affiliations:** 1 Department of Biochemistry, McGill University, Montreal, H3G1Y6 Quebec, Canada; 2 Institute of Parasitology, McGill University, Ste. Anne de Bellevue, H9X 3V9 Quebec, Canada; 3 Institute of Parasitology, Biology Centre and Faculty of Science, University of South Bohemia, 37005 České Budějovice (Budweis), Czech Republic

**Keywords:** trypanosome, RNA editing, MRP1/2, inhibitor, RNA-binding protein, RNA editing initiation

## Abstract

The RNA editing core complex (RECC) catalyzes mitochondrial U-insertion/deletion mRNA editing in trypanosomatid flagellates. Some naphthalene-based sulfonated compounds, such as C35 and MrB, competitively inhibit the auto-adenylylation activity of an essential RECC enzyme, kinetoplastid RNA editing ligase 1 (KREL1), required for the final step in editing. Previous studies revealed the ability of these compounds to interfere with the interaction between the editosome and its RNA substrates, consequently affecting all catalytic activities that comprise RNA editing. This observation implicates a critical function for the affected RNA binding proteins in RNA editing. In this study, using the inhibitory compounds, we analyzed the composition and editing activities of functional editosomes and identified the mitochondrial RNA binding proteins 1 and 2 (MRP1/2) as their preferred targets. While the MRP1/2 heterotetramer complex is known to bind guide RNA and promote annealing to its cognate pre-edited mRNA, its role in RNA editing remained enigmatic. We show that the compounds affect the association between the RECC and MRP1/2 heterotetramer. Furthermore, RECC purified post-treatment with these compounds exhibit compromised in vitro RNA editing activity that, remarkably, recovers upon the addition of recombinant MRP1/2 proteins. This work provides experimental evidence that the MRP1/2 heterotetramer is required for in vitro RNA editing activity and substantiates the hypothesized role of these proteins in presenting the RNA duplex to the catalytic complex in the initial steps of RNA editing.

## INTRODUCTION

Most mitochondrial transcripts in kinetoplastids undergo a unique post-transcriptional maturation process known as RNA editing, which is essential for parasite energy generation and viability of both insect procyclic form (PF) and mammalian bloodstream form (BF), and is thus considered a suitable drug target (Schnaufer et al. 2001; Salavati et al. 2012). A multiprotein RNA editing core complex (RECC; a.k.a. the ∼20S editosome) mediates the RNA editing process by coordinated catalytic activities, including endonuclease cleavage, uridylate (U) insertion, U deletion, and RNA ligation, as specified by complementary guide RNA molecules (gRNAs) (Blum et al. 1990; Sturm and Simpson 1990; Simpson et al. 2000, 2003; Osato et al. 2009; Aphasizhev and Aphasizheva 2011).

Several naphthalene-based sulfonated compounds, such as C35 and MrB (also referred to as V2 and V4, respectively [Durrant et al. 2010]), competitively inhibit the auto-adenylylation and, consequently, the ligation activities of the recombinant kinetoplastid RNA editing ligase 1 (rKREL1) protein from *T. brucei*; the enzyme responsible for the last step in RNA editing (Amaro et al. 2008; Durrant et al. 2010; Moshiri et al. 2011). C35 also inhibits the auto-adenylylation activity of a related RNA editing ligase, KREL2 (Moshiri et al. 2011), as both ligases have considerable sequence conservation (Worthey et al. 2003). The catalytic mechanism of the RNA editing ligases is identical to ATP-dependent DNA ligases found in eukaryotes, viruses, and eubacteria (Doherty and Suh 2000). Catalysis follows a three-step process, with (a) activation of the ligase in the first step by the formation of a covalent AMP—ligase intermediate (auto-adenylylation), releasing pyrophosphate. Next, (b) the ligase transfers the AMP to the 5′ phosphate of a nicked RNA, and (c) terminates ligation by forming a phosphodiester bond between the 3′ hydroxyl and the 5′ adenylylated phosphate, releasing the AMP (Doherty and Suh 2000). KREL1 and KREL2 are proposed to ligate the edited sites post U-deletion and U-insertion editing, respectively (Huang et al. 2001; Schnaufer et al. 2003). However, knockout studies showed that only KREL1 is essential for in vivo editing and survival of the parasites (Schnaufer et al. 2001; Drozdz et al. 2002; Gao and Simpson 2003). In the context of purified editing extracts, these compounds inhibit the in vitro RNA-binding capacity of the editosomes, consequentially affecting all in vitro RNA editing activities, suggesting an alternative mode of inhibition (Moshiri et al. 2011, 2015). Moreover, ligase auto-adenylylation enhancement is an unexpected effect of these compounds that is a result of inaccessible endogenous “ligatable” RNA molecules to which these ligases normally deadenylylate to (Moshiri et al. 2011). However, the exact mechanism of action of these compounds in the context of editosomes remained to be determined.

The RECC works with other multiprotein complexes including the RNA Editing Substrate binding Complex (RESC; a.k.a. the mitochondrial RNA binding 1 [MRB1] complex), constituting the RNA editing holoenzyme (Aphasizheva et al. 2014; Read et al. 2016). The RESC consists of the gRNA binding complex (GRBC) and the RNA editing mediator complex (REMC). The GRBC is implicated in gRNA stability and initiation of RNA editing (Weng et al. 2008; Hashimi et al. 2009; Ammerman et al. 2011, 2013), while REMC is critical for 3′ to 5′ progression (Fisk et al. 2008; Ammerman et al. 2010).

Formerly known as the gRNA binding proteins gBP21 and gBP25, the mitochondrial RNA binding proteins 1 and 2 (MRP1/2) form a ∼100 kDa stable heterotetrameric complex (Blom et al. 2001; Aphasizhev et al. 2003; Schumacher et al. 2006; Zikova et al. 2008) that associates with the RECC and the RESC in a RNA-dependent manner at low salt concentrations (Allen et al. 1998; Aphasizhev et al. 2003; Weng et al. 2008; Osato et al. 2009). In vitro biochemical analyses of recombinant MRP1/2 and their sensitivity to increasing salt concentrations support the electrostatic nature of their interaction with gRNA (Koller et al. 1997; Schumacher et al. 2006). In addition to its gRNA binding activity, this complex also promotes gRNA melting of stem loop I and gRNA : pre-mRNA annealing (Muller et al. 2001; Muller and Goringer 2002; Aphasizhev et al. 2003; Schumacher et al. 2006). RNA interference analysis of MRP2, either alone or together with MRP1, results in cell growth defects and decreased abundance of edited mRNAs (such as CyB and RPS12) and, intriguingly, never edited mRNAs (Vondruskova et al. 2005; Fisk et al. 2009), implying that MRP1/2 function may extend beyond mitochondrial transcripts requiring RNA editing. Knockdown studies revealed an effect on the assembly and functionality of the respiratory complexes with a significant decrease in the mitochondrial membrane potential, implying repression of editing in vivo (Zikova et al. 2006). Moreover, immunodepletion of MRP1 from mitochondrial fractions active in RNA editing leads to suppression of in vitro editing (Lambert et al. 1999). Recent in vivo cross-linking analysis reports MRP1/2's role in the editing of minimally edited mRNAs, resulting from a correlation between the binding patterns of MRP1 and the RESC component, MRB8170, across minimally edited mRNAs (Dixit and Lukeš 2018). While the general perception on the role of this heterotetramer remains in RNA matchmaking and annealing, its exact role and function in RNA editing is unclear.

Here, we identify the MRP1/2 proteins as targets of C35 and with its help we show that MRP1/2 inhibition abrogates in vitro RNA editing activity, implicating the MRP1/2 accessory complex in RNA editing initiation as proposed earlier.

## RESULTS AND DISCUSSION

### C35 inhibits the RNA binding activity of MRP1/2 proteins

**FIGURE 1.**
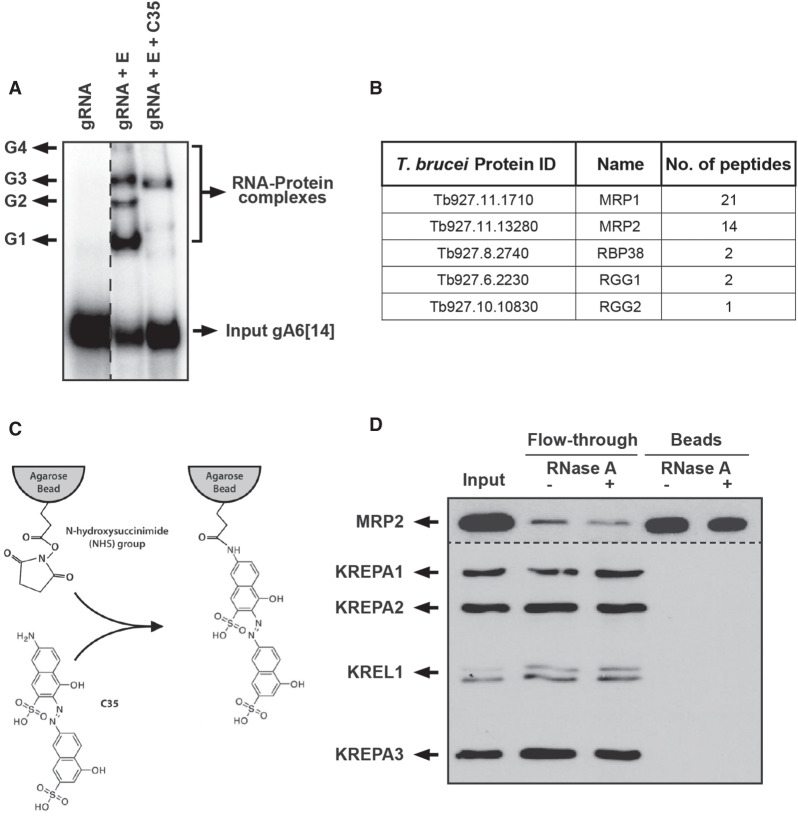
MRP1/2 are found in the G1 RNP and bind directly to C35. (*A*) C35-treated and untreated editosome proteins (E) were incubated with ^32^P-labeled gA6[14] RNA and resolved on 4% (w/v) TBE gel. Protein-bound RNA is indicated as G1–G4 RNP complexes. (*B*) The RNA-binding proteins identified in the G1 complex by tandem mass spectrometry. The corresponding protein identification numbers in *T. brucei* and the number of peptide hits are shown. (*C*) Covalent conjugation of C35 to the NHS-activated beads through its amine group. (*D*) Western blot analysis with antibodies against MRP2 (*top* panel) and KREPA1, KREPA2, KREL1, and KREPA3 (*lower* panel) analyzing mitochondrial lysate (input), unbound (flow-through), and bound proteins with and without RNase A treatment.

As shown previously, C35 targets and inhibits the formation of the ribonucleoprotein (RNP) complexes on an electro-mobility shift assay (EMSA, G1–G4) (Fig. 1A; Moshiri et al. 2011). We analyzed the content of the most affected RNP (G1) using mass spectroscopy and observed that the MRP1/2 proteins predominantly populate it. This is in accordance with previous observations where incubation with antibodies specific to MRP1 resulted in a supershift of the G1 RNP complex on an EMSA (Allen et al. 1998). While this observation indicated that these proteins were the likely targets for C35 in the G1 RNP, we cannot rule out the possibility of other RNA binding proteins also being targeted, namely TbRBP38, TbRGG1, and TbRGG2 that are present at much lower abundance. Figure 1B summarizes the results obtained from this mass spectrometric study. To validate MRP1/2 as bona fide targets of C35, the compound was covalently conjugated to agarose beads *via* a N-hydroxysuccinimide linker that binds to the primary amine group on the compound. These beads were used as bait to precipitate the potential target proteins from the *T. brucei* mitochondrial lysate in the presence or absence of RNase A, since RECC interaction with MRP1/2 heterotetramer is RNase sensitive (Aphasizhev et al. 2003; Osato et al. 2009). Western blotting with antibodies of MRP2 and four RECC proteins (KREPA1, KREPA2, KREL1, and KREPA3) indicated that MRP2 potentially binds directly to C35, as that pull-down is unaffected in the presence of RNase A. In contrast, the RECC proteins (especially KREL1) do not bind at all (Fig. 1D). A majority of the interactions between the MRP1/2 protein complex and their target RNA are electrostatic in nature, as these proteins are rich in arginine residues with a theoretical pI > 9 (Koller et al. 1997; Schumacher et al. 2006). On the other hand, C35, with its sulfonate groups, is highly electronegative (Durrant et al. 2010), suggesting its interaction with MRP1/2 to also be governed by electrostatic interactions. So, as C35 occupies the RNA-binding regions of the MRP1/2 proteins, they displace any bound RNA and, consequently, the RECC complexes, thereby affecting the association between the two complexes.

**FIGURE 2.**
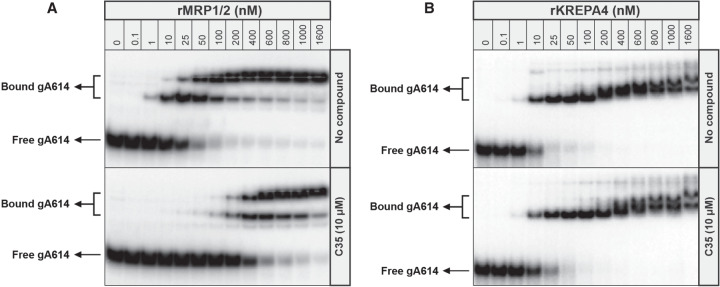
C35 affects the RNA binding activity of recombinant MRP1/2 proteins. Increasing concentrations of the respective proteins were incubated with ^32^P-labeled gA6[14] gRNA in the absence and presence of C35 at 10 µM: (*A*) rMRP1/2 proteins and (*B*) rKREPA4. The positions of free gA6[14] and protein-bound gA6[14] as resolved on the native TBE gels are indicated.

Using EMSAs, we next validated C35's effect on the gRNA binding activities of coexpressed recombinant MRP1/2 and KREPA4 (Fig. 2); KREPA4 is an integral RECC protein known to interact with gRNA and is used as a control protein (Salavati et al. 2006; Kala and Salavati 2010). Increasing concentrations of the purified recombinant proteins (0.1–1600 nM) were incubated with a fixed amount of labeled gA6[14] gRNA in the absence or presence of 10 µM C35. As seen in Figure 2A, untreated MRP1/2 shows the characteristic pattern of the gRNA-MRP1/2 assembly (Zikova et al. 2008): formation of two different complexes corresponding to one or two gRNA molecules bound, which is decreased in the presence of C35, requiring a higher concentration of MRP1/2 to overcome the inhibition. On the other hand, the interaction between KREPA4 and gA6[14] is effectively unperturbed by C35 (Fig. 2B). These data demonstrate that C35 directly binds to and inhibits the RNA binding activity of MRP1/2.

### Purified editosomes with lower MRP1/2 abundance exhibit increased susceptibility to C35 and MrB inhibition

To assess the effect of MRP1/2 inhibition in the context of in vitro RNA editing activities, functional editosomes with differing abundances of MRP1/2 were prepared using tandem affinity purification (TAP) of tagged KREL1. The TAP-tag fused to KREL1 contains two affinity tags, a protein A domain and a calmodulin-binding peptide, separated by a TEV protease cleavage site. This system allows for sequential purification of the tagged RECC through IgG agarose (TEV protease elution; “TEV eluate”) and calmodulin affinity resins (EGTA elution; “Calmodulin eluate”). The resulting eluates were analyzed against the KREPA1, KREPA2, KREPA3, and KREL1 antibodies on a western blot, and eluate volumes corresponding to equal intensities were used in further experiments (data not shown). Equal band intensities on this western blot correlated to 90 ng/µL of total protein concentration in the TEV eluate versus 35 ng/µL of total protein concentration in the Calmodulin eluate, as estimated through Bradford quantification.

**FIGURE 3.**
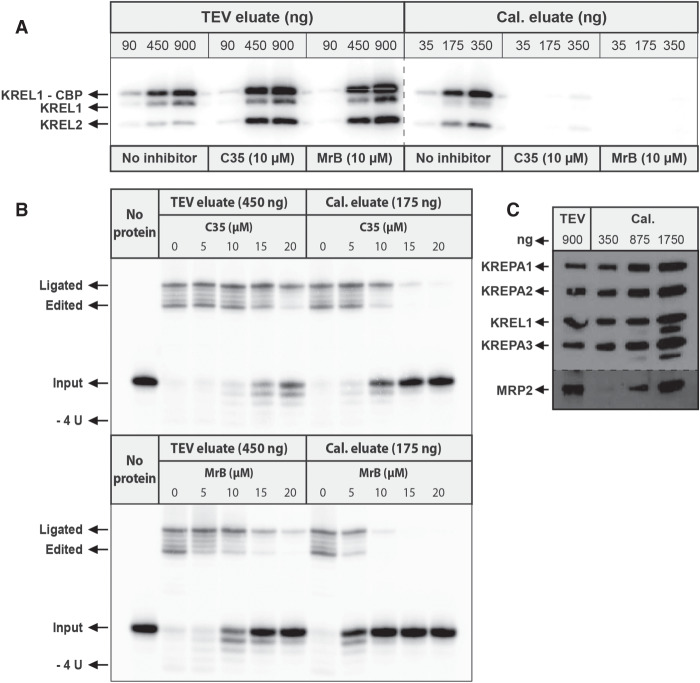
C35 and MrB inhibition of ligase auto-adenylylation and in vitro “precleaved” deletion in KREL1–TEV and Calmodulin eluates are correlated with MRP1/2 abundance. (*A*) KREL1 and KREL2 auto-adenylylation activities with various amounts of the KREL1–TEV and calmodulin eluates in the absence or presence of C35 and MrB (at 10 µM). Endogenous KREL1, KREL2, and KREL1-calmodulin fusion (KREL1-CBP) proteins are indicated. (*B*) In vitro “precleaved” deletion activities of both eluates in increasing amounts of C35 and MrB. (*C*) Western blot analysis showing the amount of MRP2 proteins in the two protein preparations. The eluates were precipitated with methanol for the purpose of concentrating them, prior to loading on the gel.

Ligase auto-adenylylation in the TEV eluate was only affected at a lower protein amount tested (90 ng) and enhanced at higher amounts (450 and 900 ng) (Fig. 3A), as observed previously (Moshiri et al. 2011). As this enhancement was earlier proposed to be a consequence of losing accessible (“ligatable”) endogenous RNA, inhibition of MRP1/2 RNA interaction by the compounds indicates that these processes could be related. In other words, the compounds added at 10 µM were sufficient for inhibiting the KRELs in 90 ng of TEV eluate, but in larger quantities of TEV eluate, and therefore larger quantities of MRP1/2, the compounds were likely absorbed by MRP1/2 displacing the bound endogenous RNA off the MRP1/2 and RECC complexes and enhancing ligase auto-adenylylation. On the other hand, the compounds inhibit ligase auto-adenylylation in all quantities of the calmodulin eluate tested (Fig. 3A), as it contains approximately fivefold less MRP1/2 proteins than in the TEV eluate as estimated from band intensities on the western blot using ImageJ software (Fig. 3C). Consistently, the TEV eluate appeared more resistant to inhibition in the “precleaved” deletion assay, requiring U-deletion and ligation activities (Fig. 3B). These data indicate that the MRP1/2 complex preferentially binds to the compounds, limiting inhibition of the ligase in the TEV eluate.

### C35 and MrB weaken the association between RECC and MRP1/2 and compromise in vitro editing

**FIGURE 4.**
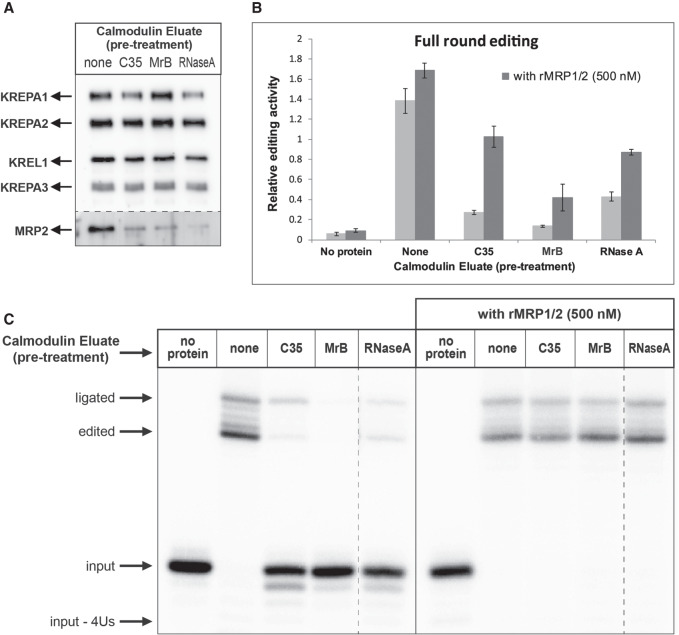
C35 and MrB disengage MRP1/2 association with RECC and suppress in vitro RNA editing activity. KREL1 Calmodulin eluates were prepared after treatment with C35, MrB, and RNase A while binding to the Calmodulin affinity resin. (*A*) Western blot analysis against KREPA1, KREPA2, KREL1, and KREPA3 antibodies in the *top* panel and against MRP2 antibody in the *bottom* panel. Each lane contains ∼900 ng of the respective eluate. (*B*) “Full-round” and (*C*) “precleaved” RNA editing activities of these eluates in absence and presence of rMRP1/2 (500 nM).

To determine if inhibition of the MRP1/2 complex by the compounds displaces the heterotetramer off the RECC, KREL1 TEV eluate was bound to the Calmodulin Affinity resin in the presence of C35, MrB (20 µM each), or RNase A (0.1 mg/mL) as a control. The EGTA elutions were performed after adequate washes to eliminate contaminating inhibitor or RNase A in the eluates. While western blots revealed a considerable loss in associated MRP2 in the eluates pretreated with the compounds and RNase A (Fig. 4A), these eluates also suffered from reduced in vitro editing activities in “full-round” and “precleaved” assays (Fig. 4B,C). This observation is concurrent with a previous study that shows suppression of in vitro editing activity upon immunodepletion of MRP1 (Lambert et al. 1999). Remarkably, their activities are recovered upon the addition of the recombinant (r) MRP1/2 proteins at ∼500 nM (Fig. 4B,C). The required rMRP1/2 concentration was determined through 10-fold serial dilutions in the “full-round” FRET-based editing assay, tested with C35 and MrB pretreated calmodulin eluates (Supplemental Fig. S1 in Supplemental Information).

**FIGURE 5.**
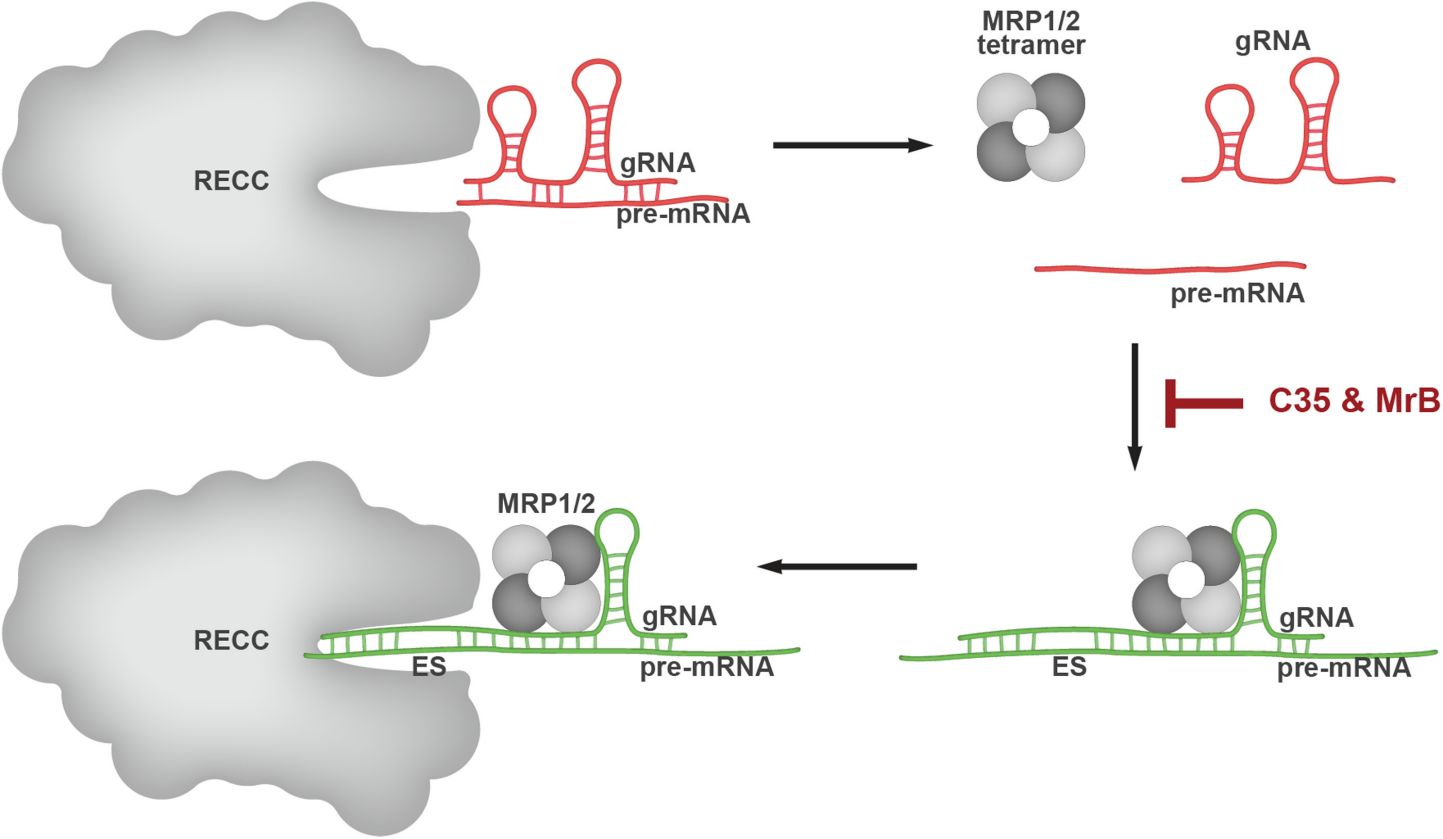
Model representing role of the MRP1/2 heterotetramer in RNA editing. The RECC or the editosome is incapable of initiating editing and requires the RNA annealing activity of the MRP1/2 heterotetramer for presenting the “editable” RNA duplex to the catalytic complex. C35 and MrB interfere with the RNA-binding capacity of MRP1/2, and therefore interfere with the RNA editing capacity of the core complex. ES indicates editing site.

These data provide compelling experimental evidence that validates the presumed role of MRP1/2 heterotetramer in trypanosomatid RNA editing initiation. The MRP1/2 complex acts potentially through its gRNA/pre-mRNA annealing activity to present the RNA duplex in a form that is accessible to the RECC, which can be inhibited by the sulfonated compounds C35 and MrB, as modeled in Figure 5 (Muller et al. 2001; Muller and Goringer 2002; Aphasizhev et al. 2003; Schumacher et al. 2006; Ammerman et al. 2008; Zikova et al. 2008). This stimulatory effect of MRP1/2 on in vitro editing is concurrent with the effect of another RNA-binding protein with annealing activity, RBP16 (Miller et al. 2006), suggesting that the RECC requires accessory annealing factors for editing. While these observations are based on experiments conducted in vitro, these accessory factors may behave similarly in vivo and will require additional experiments to substantiate this conclusion. The other major finding of this study is the inhibition susceptibility of in vitro RNA editing by negatively charged compounds, as these compounds electrostatically compete away the RNA bound to the MRP1/2 proteins. We believe this mode of action extends to the RNA editing inhibitors previously found in the pilot scale screen conducted using the library of pharmacologically active compounds (LOPAC^1280^, Sigma), namely aurintricarboxylic acid (ATA), PPNDS and NF449, that not only affect RNA–protein interaction but also all catalytic activities required in editing in vitro (Moshiri et al. 2015). Preliminary analysis against suramin indicates a similar effect (data not shown), as it contains sulfonated naphthalene groups similar to C35 and MrB, and inhibits rKREL1 auto-adenylylation (Zimmermann et al. 2016).

## MATERIALS AND METHODS

### Preparation of RNA and radiolabeling

Electrophoretic mobility shift assays were performed with synthetic gA6[14] gRNA that specifies editing of the first editing site of the ATPase subunit 6 (A6) pre-mRNA. Preparation of gA6[14] gRNA was carried out via in vitro transcription of PCR amplified DNA template as described previously (Seiwert and Stuart 1994), and radiolabeled at the 3′-terminus by incubating 50 pmol gA6[14] gRNA with 50 µCi of PcP [5′-^32^P] (3000 Ci/mmol, 10 mCi/mL) at 4°C overnight, in a total reaction volume of 20 µL, containing 50 mM Tris-HCl [pH 7.5], 10 mM MgCl_2_, 1 mM ATP, 1 mM DTT, 15% glycerol, 10% DMSO, and 10 units of T4 RNA ligase 1 (New England Biolabs). RNA substrates required for the in vitro precleaved deletion editing assay (U5-5′CL, U5-3′CLpp, and gA6[14]PC-del) were prepared as described previously (Igo et al. 2002). The 5′ fragment was radiolabeled at its 5′ terminus by incubating 50 pmol of the RNA with 50 µCi of ATP [γ-^32^P] (3000 Ci/mmol, 10 mCi/mL) at 37°C for 1 h, in a 10 µL reaction, containing 70 mM Tris-HCl [pH 7.6], 10 mM MgCl_2_, 5 mM DTT, and 10 units of T4 polynucleotide kinase (New England Biolabs). The radiolabeled RNA was PAGE-purified on a 9% (w/v) 7 M urea denaturing gel and quantified by scintillation counting. RNA substrates required in the in vitro FRET-based editing assay (pre-A6Rbz, gA6Rbz, gA6Rbz-comp, and FRET substrate) were prepared as described previously (Moshiri and Salavati 2010; Moshiri et al. 2014).

### Preparation of active proteins for functional studies

The crude mitochondrial extract was prepared through hypotonic lysis of the procyclic stage *T. brucei* (strain IsTaR 1.7A) cells grown in SDM-79 media to late log phase (∼2 × 10^7^ cells/mL). Following glycerol gradient sedimentation (10%–30%) of the crude mitochondrial lysate, 500 µL fractions containing active in vitro RNA editing activity (F10–F12) were isolated (Panigrahi et al. 2003; Moshiri et al. 2014), pooled and concentrated to 50 µL using Amicon Ultra 0.5 mL centrifugal filters. Immunoprecipitated editosomes were also prepared from KREL1-TAP 29.13 procyclic cells (Moshiri et al. 2011), wherein both IgG column (TEV protease release; named TEV eluate in this study) and calmodulin column preparations (EGTA release; named calmodulin eluate) were obtained and stored at −80°C. Recombinant (r) MRP1/2 and KREPA4 proteins were purified as done previously (Zikova et al. 2008; Kala and Salavati 2010). To examine the presence of MRP1/2 in TEV and calmodulin eluates of KREL1-TAP, varying protein amounts from methanol precipitated eluates were analyzed by western blotting using the polyclonal antibody against MRP2, as described previously (Vondruskova et al. 2005).

### Electrophoretic mobility shift assays (EMSA)

To identify the proteins affected by C35 treatment, editosomes partially purified by glycerol gradient sedimentation of the mitochondrial extract (concentrated fractions F10–F12) were used. An amount of 15 µL of this preparation (10 µg/µL) was incubated with 5 pmol of unlabeled gA6[14] RNA and 0.5 pmol (3 × 10^4^ CPM) of the 3′ end-labeled gA6 [14] RNA in a total 20 µL of a mixture containing 20 mM Tris-HCl [pH 7.6], 150 mM KCl, 5 mM MgCl_2_, 100 µg/mL BSA, 10% glycerol, 1 mM DTT, and 20 units of RNasin ribonuclease inhibitor (Promega) for 30 min at room temperature. The RNA substrates were heated at 95°C for 5 min and cooled at room temperature prior to assembling the reactions. Samples were mixed with gel loading solution (0.25% bromophenol blue, 0.25% xylene cyanol, and 30% glycerol) and run on 4% (w/v) native TBE gels as per the protocol (Kala and Salavati 2010). The gel run with unlabeled gA6[14] was stained using a colloidal Coomassie solution (BioRad), and the staining pattern was correlated with the RNA–protein complexes in the phosphorimage of the gel run with radiolabeled gA6[14]. The bands corresponding to the G1–G4 complex were excised from the gel run with unlabeled gA6[14] and analyzed by mass spectrometry.

To monitor the inhibitory effect of C35 on RNA binding proteins, increasing concentrations (0.1–1600 nM) of purified rMRP1/2 and rKREPA4 were first incubated in the presence and absence of 10 µM C35 on ice for 10 min. These mixtures were then incubated with 0.5 pmol labeled gA6[14] for 30 min at room temperature as done above, run on 10% (w/v) native TBE gels (BioRad) and visualized by phosphorimaging.

### Auto-adenylylation and in vitro RNA editing assays

KREL1 auto-adenylylation assays were performed using various amounts of KREL1-TAP TEV and calmodulin eluates corresponding to 0.09, 0.45, and 0.9 µg and 0.035, 0.175, and 0.35 µg of total protein, respectively. The eluates were incubated in a 20 µL reaction mixture containing 12.5 mm HEPES (pH 7.9), 25 mm KCl, 5 mm magnesium acetate, 0.25 mm DTT, 40 nm [α-^32^P] ATP (3000 Ci/mmol, 10 mCi/mL), 0.1% (w/v) Triton X-100, and 5% (v/v) of DMSO (control) or 1 µL of 200 µM C35 or MrB (dissolved in DMSO; corresponding to 10 µM in a 20 µL reaction), as described elsewhere (Moshiri et al. 2011). The proteins were preincubated with the respective compounds on ice for 5 min prior to reaction assembly. The adenylylation reactions were allowed to run at 28°C for 10 min, stopped by the addition of SDS loading buffer and resolved on 10% SDS-PAGE gel. Radiolabeled proteins were detected by phosphorimaging.

In vitro precleaved RNA editing assays were performed following procedures from literature (Igo et al. 2002). RNA substrates required for the precleaved deletion assay, [γ-^32^P] U5-5′CL, U5-3′CLpp, and gA6[14]PC-del were mixed in a ratio of 0.25:1:0.5 pmol, heated at 65°C for 2 min and cooled down to room temperature. Editing reactions were prepared by assembling 5 µL of TEV eluate (0.45 µg of total protein) or 5 µL of calmodulin eluate (0.175 µg of total protein) with the RNA mixture, in a 20 µL solution containing 25 mM HEPES [pH 7.9], 10 mM Mg(OAc)_2_, 1 mM EDTA, 5 mM CaCl_2_, 10 µM ATP, 0.1% Triton X-100 (w/v), 16.7 ng/µL Torula yeast RNA, 15 units of RNasin plus ribonuclease inhibitor, and 0.5 mM DTT. rMRP1/2 was added in the reactions as indicated. The editing reactions were allowed to proceed for 3 h at 28°C and stopped by the addition of 2 µl of 260 mM EDTA-2.5% sodium dodecyl sulfate mixture. RNA was then extracted using phenol:chloroform:isoamyl alcohol (25:24:1), precipitated and resuspended in 10 M urea dye before running onto 15% (w/v) 7 M urea denaturing polyacrylamide gel and visualized by phosphorimaging. Full-round FRET-based RNA editing assay was performed identically as described previously (Moshiri and Salavati 2010; Moshiri et al. 2014). rMRP1/2 was added in the reactions as indicated.

## SUPPLEMENTAL MATERIAL

Supplemental material is available for this article.

## Supplementary Material

Supplemental Material
