# Supplementary material for: Sulfonated inhibitors of the RNA editing ligases validate the essential role of the MRP1/2 proteins in kinetoplastid RNA editing
Source: RNA. 2020 Jul;26(7):827–35. doi: 10.1261/rna.075598.120 (PMC7297121; doi:10.1261/rna.075598.120)
Supplement: Supplemental Material [file supp_26_7_827__index.html]

Sulfonated inhibitors of the RNA editing ligases validate the essential role of the MRP1/2 proteins in kinetoplastid RNA editing — Supplemental Material 

# Sulfonated inhibitors of the RNA editing ligases validate the essential role of the MRP1/2 proteins in kinetoplastid RNA editing

## Supplemental Material

- Supplemental\_Material.pdf
