# Supplementary material for: Sulfonated inhibitors of the RNA editing ligases validate the essential role of the MRP1/2 proteins in kinetoplastid RNA editing
Source: RNA. 2020 Jul;26(7):827–35. doi: 10.1261/rna.075598.120 (PMC7297121; doi:10.1261/rna.075598.120)
Supplement: Supplemental Material [file supp_075598.120_Supplemental_Material.pdf]

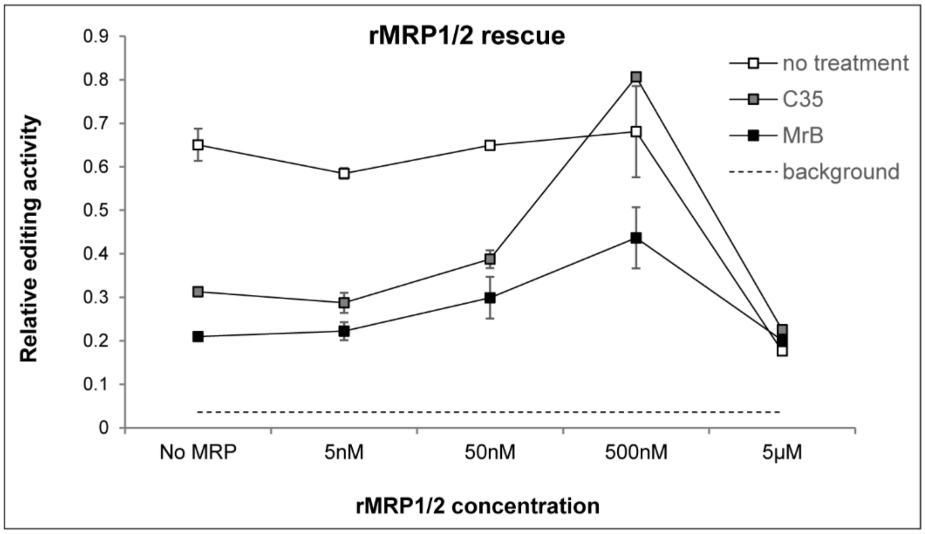

Figure S1. Determining rMRP1/2 concentration for rescuing compromised TbREL1 - Calmodulin eluates (pre-treated with C35 and MrB). rMRP1/2 was added to the FRET-based "full-round" editing assay in a tenfold serial dilution. Untreated eluate was used as a positive control.
